# Immune Cells as Mediators of Lipidome Influence on Osteoporosis: Evidence from a Mediation Analysis

**DOI:** 10.3390/diagnostics15101287

**Published:** 2025-05-20

**Authors:** Jiheng Xiao, Wei Zhou, Jiatai He, Yanbin Zhu, Yingze Zhang, Liming Xiong

**Affiliations:** 1 Department of Orthopaedics, Union Hospital, Tongji Medical College, Huazhong University of Science and Technology, Wuhan 430022, China; 2 Department of Orthopaedic Surgery, Third Hospital of Hebei Medical University, Shijiazhuang 050051, China; 3 NHC Key Laboratory of Intelligent Orthopaedic Equipment, Third Hospital of Hebei Medical University, Shijiazhuang 050051, China; 4 Department of Urology, Union Hospital, Tongji Medical College, Huazhong University of Science and Technology, Wuhan 430022, China; 5 School of Medicine, Nankai University, Tianjin 300071, China

**Keywords:** lipidome, immune cells, osteoporosis, mendelian randomization, mediating effect

## Abstract

**Background**: Although clinical studies have indicated a possible association between dyslipidemia and osteoporosis, the underlying genetic basis and mechanistic pathways remain insufficiently defined. Most prior research has concentrated on conventional lipid markers, which are prone to confounding and limit causal inference. Exploring lipidomic profiles offers a more comprehensive view of lipid metabolism and may reveal novel genetic links beyond traditional lipid traits. Additionally, alterations in immune cell function, often triggered by metabolic disturbances, may contribute to osteoporosis development; however, the potential mediating role of immune cells in the lipid–bone axis has not been systematically investigated. **Methods**: A total of 179 lipid species across 13 lipid classes were analyzed in 7174 Finnish individuals from the GeneRISK cohort. Genome-Wide Association Study (GWAS) summary statistics for osteoporosis and 731 immune cell immunophenotypes were sourced from the GWAS Catalog. A two-step, two-sample Mendelian randomization analysis, using inverse variance weighting (IVW), was conducted to explore the potential causal effects of lipids on osteoporosis and the mediating role of immune cells in the relationship between lipids and osteoporosis. **Results**: Mendelian randomization analysis indicated that triacylglycerol levels of 48:0 were possibly associated with an increased risk of osteoporosis (IVW: odds ratio [OR] 1.1320, 95% CI 1.0401–1.2321; *p* = 0.004), while triacylglycerol levels of 48:3 appeared to be associated with a reduced risk of osteoporosis (IVW: OR 0.9053, 95% CI 0.8364–0.9800; *p* = 0.014). Two statistically significant mediating effects were identified: First, IgD− CD38dim %B cells appeared to partially negatively mediate the association between triacylglycerol levels of 48:3 and osteoporosis, with a negative mediating effect of −0.00669 (95% CI: −0.0214, 0.00805), which accounted for 6.73% of the total effect. That is, the protective effect of triacylglycerol levels of 48:3 against osteoporosis was attenuated by IgD− CD38dim %B cells. Second, HLA DR++ monocytes% leukocytes also partially negatively mediated this relationship, with a mediating effect of −0.023 (95% CI: −0.0434, −0.00266), accounting for 23.2% of the total effect. This indicates that other immune cells, HLA DR++ monocytes %leukocytes, resisted the protective effect of triacylglycerol levels of 48:3 against osteoporosis, with a weakening effect stronger than that of IgD− CD38dim %B cells. **Conclusions**: Our findings contribute to the growing understanding of the potential causal relationships and shared pathogenic mechanisms between dyslipidemia and osteoporosis. The results suggest that the potential genetic effects of plasma lipid metabolites on osteoporosis may be partially down-regulated by specific kinds of immune cells.

## 1. Introduction

Bone integrity is maintained through the dynamic balance between bone resorption and formation, a physiological process known as bone homeostasis [1]. Osteoporosis, a progressive systemic skeletal disorder, results from an imbalance in this homeostatic mechanism, characterized by reduced bone mass and deterioration of bone microarchitecture. These pathological changes increase the risk of fragility fractures, posing substantial health and economic burdens, particularly in aging populations [2,3].

Emerging evidence highlights a complex and bidirectional interaction between lipid metabolism and bone health, implicating lipid species (lipidome) in the pathophysiology of osteoporosis. Studies have shown that total cholesterol (TC) and its metabolites influence osteoblast activity both in vitro and in vivo [4]. Hyperlipidemia contributes to subendothelial lipid deposition in bone vasculature, thereby impairing osteoblast differentiation and mineralization. Paradoxically, although high-density lipoprotein cholesterol (HDL-C) is considered protective in cardiovascular disease, elevated HDL-C levels have been associated with an increased risk of osteoporosis [5]. Additionally, recent lipidomics research suggested that triacylglycerols (TAGs) are not merely energy reserves but active regulators of bone metabolism [6,7]. Triglyceride-rich lipoproteins, particularly in the context of obesity or dyslipidemia, contribute to bone loss by modulating inflammatory pathways and altering MSC differentiation. Intriguingly, the study also proposed that low to moderate levels of TAGs may exert protective effects on bone mass, highlighting a possible dose-dependent dual role of triglycerides in skeletal health. Furthermore, a meta-analysis encompassing 33 studies indicated that statin use is associated with a reduced risk of hip fractures, reinforcing the clinical relevance of lipid-lowering therapies in bone health [8].

Beyond direct metabolic effects, dyslipidemia has been implicated in the promotion of chronic low-grade inflammation [9,10]. Immune cells, such as macrophages, dendritic cells, T and B lymphocytes, and neutrophils, mediate the inflammatory responses that influence skeletal remodeling. The immune system, comprising diverse cell types, cytokines, and signaling pathways, is increasingly recognized as a central regulator of bone metabolism. Recent population-based studies have shown that elevated levels of granulocyte-macrophage colony-stimulating factor (GM-CSF), predominantly secreted by B lymphocytes, are associated with increased osteoporosis risk, highlighting the role of immune dysregulation in bone loss [11,12]. While these findings underscore a strong immunological component in osteoporosis, the specific immune mechanisms that bridge lipid metabolism and bone deterioration remain largely undefined.

To date, no studies have systematically investigated whether immune cell populations affected by lipidomic alterations exert a causal influence on osteoporosis. Given the intertwined roles of lipids and immunity in skeletal biology, elucidating these mediating pathways is of critical importance. Mendelian randomization (MR), a genetic epidemiological method using single nucleotide polymorphisms (SNPs) as instrumental variables, offers a robust framework for inferring causality while minimizing confounding and reverse causation [13].

In this study, we employed a two-sample, bidirectional MR design combined with mediation analysis to investigate the causal relationships between lipidomic profiles and osteoporosis. We further explored the potential mediating role of immune cells in this association, aiming to provide novel mechanistic insights into the immunometabolic regulation of bone health.

## 2. Methods

### 2.1. Study Design

**Figure 1:**
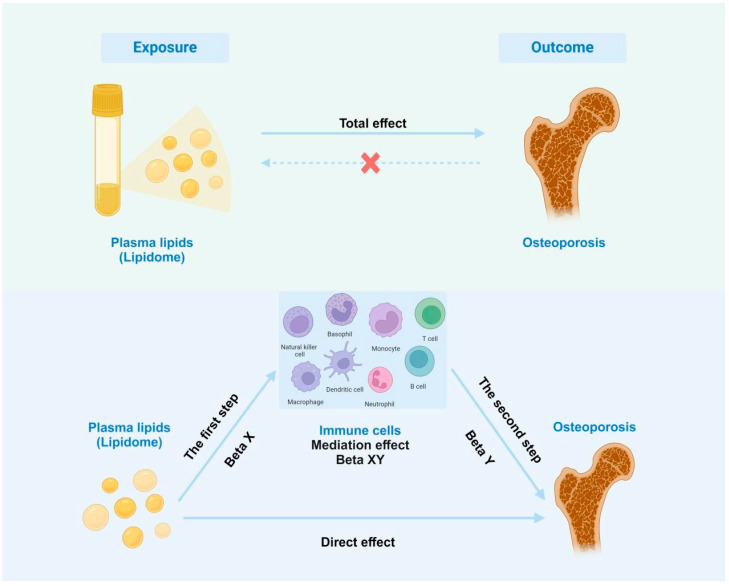
Schematic of two-sample, two-step Mendelian randomization. Beta X, the causal effect of the lipidome on immune cells; Beta Y, the causal effect of immune cells on osteoporosis; Beta XY, the role of immune cells in mediating the effect of the lipidome on osteoporosis.

(I) The lipidome and osteoporosis were subjected to a two-sample Mendelian randomization analysis to select genetic variants that are representative of plasma lipids and have no reverse causality. (II) Immune cells with strong correlations with osteoporosis and the lipidome were screened as potential mediators using a two-step Mendelian randomization study, assessing the causal effects of lipids on immune cells and immune cells on osteoporosis, respectively, and estimating the mediating effect of immune cells in the causal relationship between lipids and osteoporosis. The scheme is summarized in Figure 1.

### 2.2. GWAS Data for the Lipidome

GWAS summary statistics for the lipidome include 179 lipid species from 13 lipid classes in 7174 Finnish individuals from the GeneRISK cohort, which were acquired from the GWAS Catalog (https://www.ebi.ac.uk/gwas/downloads/summary-statistics, accessed on 12 May 2024. First author: Ottensmann L; PubMed ID: 37907536) [14].

### 2.3. GWAS Data for Osteoporosis (OP)

GWAS summary statistics for osteoporosis were obtained from the FinnGen consortium R10 release data (https://r10.finngen.fi/pheno/M13_OSTEOPOROSIS, accessed on 12 May 2024). The OP GWAS dataset ID is finngen_R10_M13_OSTEOPOROSIS. This GWAS included 399,054 Finnish adult subjects and consisted of 8017 cases and 391037 controls. Osteoporosis is classified according to the International Classification of Diseases (ICD-10 M80 M81 M82, ICD-9 733(0-1), ICD-8 7230 72391). It is characterized by a reduction in bone mass, cortical thickness, and the number and size of trabeculae in cancellous bone, with normal bone composition, leading to an elevated fracture risk. The condition includes primary osteoporosis (Type 1, postmenopausal; Type 2, age-related; and idiopathic, which may affect adolescents, premenopausal women, and middle-aged men) and secondary osteoporosis, which is linked to an identifiable cause of bone loss.

### 2.4. GWAS Data for Immune Traits

GWAS summary statistics for each immune cell characteristics are publicly available from the GWAS Catalog, accession numbers from GCST0001391 (https://www.ebi.ac.uk/gwas/studies/GCST0001391, accessed on 12 May 2024) to GCST0002121 (https://www.ebi.ac.uk/gwas/studies/GCST0002121, accessed on 12 May 2024) [15]. This study incorporated a comprehensive panel of 731 immune-related phenotypes categorized into four distinct types: absolute cell counts (*n* = 118), relative cell proportions (*n* = 192), surface antigen expression intensities measured via the median fluorescence intensity (MFI, *n* = 389), and morphological descriptors (*n* = 32). These traits encompassed a wide range of immune subpopulations, including B lymphocytes, conventional dendritic cells (cDCs), T cell subsets at various maturation stages, monocytes, myeloid cells, and regulatory T cells, as assessed through TBNK and lineage-specific immunophenotyping panels. Notably, morphological features were available for the cDC and TBNK subgroups. A total of 3757 individuals of European ancestry were included. Genotyping was conducted using high-density SNP arrays, and imputation was performed against a Sardinian whole-genome reference panel to yield approximately 22 million variants. Statistical analyses adjusted for key covariates, including age, sex, self-reported ancestry, and occupation, to control for population stratification and environmental influences.

Importantly, datasets used for immune traits, lipidomic profiling, and osteoporosis outcomes were obtained from entirely independent cohorts. Care was taken to ensure the complete absence of participant overlap across the exposure, mediator, and outcome datasets. This design minimized potential bias arising from sample duplication and strengthened the validity of causal inference derived from the Mendelian randomization analyses. Detailed accession information for each dataset is available in the public repositories.

### 2.5. Instrumental Variable Selection

In order to obtain significantly associated single-nucleotide polymorphisms (SNPs), the genome-wide significance threshold was set to *p* < 5 × 10^−8^ in this study. Second, to ensure the independence of each SNP, the eligible SNPs were retained according to the linkage disequilibrium (LD) condition (R^2^ = 0.001, kb = 10,000). Subsequently, the outliers in IV were further cleaned up using the outlier correction method in the MR -PRESSO model. Finally, weak IVs with F-statistics of less than 10 were excluded to ensure the validity of the IVs [16]. The specific formula for calculating the F-statistic is F = β^2^/SE^2^, where β is the allele effect value (the effect value corresponding to each genetic variant k locus, is the beta value) and SE is the standard error.

Choosing SNPs to study the relationship between the lipidome and osteoporosis requires meeting three key assumptions of Mendelian randomization: (I) The genetic instrumental variables (IVs) demonstrate a strong and robust association with the lipidome traits, satisfying the relevance assumption of Mendelian Randomization. (II) The lipidome IVs fulfill the independence assumption of Mendelian Randomization, as they show no association with any known or potential confounders. (III) The IVs do not have a direct impact on osteoporosis, and their influence on the outcome is solely through the lipidome.

### 2.6. Statistical Analysis

In this study, we used multiple methods to evaluate the causative association between 179 lipid species with osteoporosis and 731 immunophenotypes by the “Two Sample MR” package (version 0.6.1), including inverse-variance weighted (IVW), MR-Egger, weighted median-based, weighted mode, and simple mode techniques. If there is no horizontal pleiotropy, the IVW results will be unbiased. Cochran *Q* statistics were used to quantify the heterogeneity of the IVs, with a *p*-value less than 0.05 indicating its presence. We generated scatter plots to show that the results were not affected by outliers and funnel plots to demonstrate the robustness of the correlation and the absence of heterogeneity, in order to present the findings of the Mendelian randomization (MR) analysis. Furthermore, this study conducted leave-one-out analysis to assess the sensitivity of the results, omitting each instrumental SNP in turn.

To explore the potential causal pathway linking lipid metabolism to osteoporosis via immune cell mediation, two-step Mendelian randomization (MR) analysis was employed. In the first step, the inverse-variance weighted (IVW) method was used to estimate the causal effect of lipid-related metabolites (the lipidome) on immune cell traits (denoted as Beta X). In the second step, the causal effect of immune cells on osteoporosis risk (Beta Y) was similarly calculated. The mediated effect (Beta XY) was derived using the product-of-coefficients approach, expressed as Beta XY = Beta X × Beta Y, thereby quantifying the extent to which immune cells mediate the lipidome–osteoporosis relationship.

The effect estimates were reported as odds ratios (ORs), with corresponding 95% confidence intervals (95% CI). Statistical significance was defined as *p* < 0.05. To mitigate the risk of false positives due to multiple comparisons, Bonferroni correction was applied; associations with *p*-values below the nominal threshold but above the corrected significance level (0.05/n, where n is the number of tests), were interpreted as suggestive but not definitive evidence of causality. All analyses were performed in R 4.3.3 software (http://www.Rproject.org).

## 3. Results

### 3.1. Exploration of the Causal Effect of Lipidome on Osteoporosis

**Figure 2:**
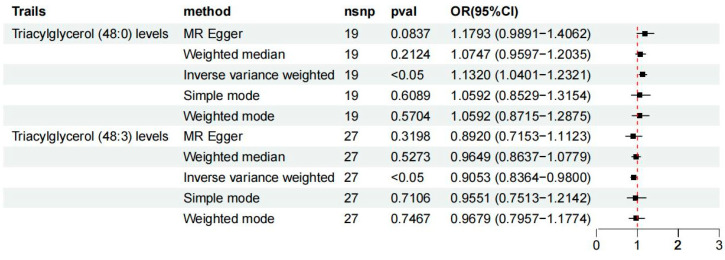
Causal effects of lipidome on osteoporosis. Triacylglycerol levels of 48:0 were linked with increased osteoporosis risk (IVW: odds ratio [OR] 1.1320, 95% confidence interval [CI] 1.0401–1.2321; *p* = 0.004). Triacylglycerol levels of 48:3 were linked with decreased osteoporosis risk (IVW: odds ratio [OR] 0.9053, 95% confidence interval [CI] 0.8364–0.9800; *p* = 0.014). CI, confidence interval; OR, odds ratio; SNP, single nucleotide polymorphism.

**Figure 3:**
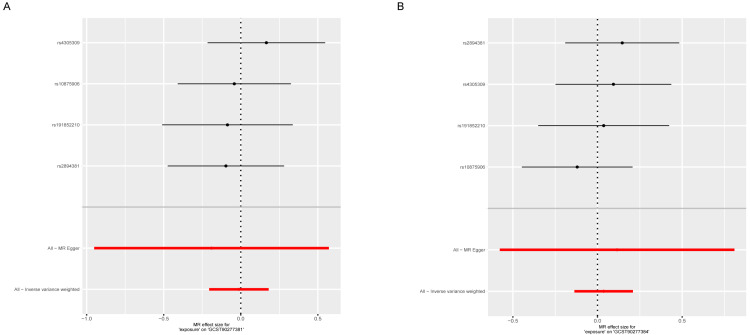
Reverse Mendelian randomization analysis assessing the causal effects of osteoporosis on plasma lipid metabolites (lipidome). (**A**,**B**) No statistically significant associations were observed when osteoporosis was used as the exposure and lipidomic traits as the outcomes.

To investigate the potential causal relationship between plasma lipid metabolites and osteoporosis, a two-sample Mendelian MR was employed, with the inverse-variance weighted (IVW) method serving as the primary analytic approach. To ensure the robustness and reliability of the identified associations, results were retained only if they satisfied the following stringent criteria: (1) a IVW-derived *p*-value of <0.05; (2) concordant effect directions across all five MR models; and (3) the absence of directional pleiotropy, as indicated by the MR-Egger intercept (*p* > 0.05). Based on these filtering standards, a total of 19 single nucleotide polymorphisms (SNPs) were identified as valid instrumental variables for triacylglycerol levels of 48:0, while 27 SNPs were selected for triacylglycerol levels of 48:3, respectively. After multiple test adjustment based on the statistical analysis, there were two suggestive lipidomes identified, both of which were in the triacylglycerol panel. Between them, triacylglycerol levels of 48:0 were linked with increased osteoporosis risk (IVW: odds ratio [OR] 1.1320, 95% confidence interval [CI] 1.0401–1.2321; *p* = 0.004). However, triacylglycerol levels of 48:3 were linked with decreased osteoporosis risk (IVW: odds ratio [OR] 0.9053, 95% confidence interval [CI] 0.8364–0.9800; *p* = 0.014) (Figure 2, Supplementary Table S1). Then, we conducted reverse mendelian randomization analysis to explore the causal effects of osteoporosis on the lipidome, as shown in Figure 3A,B and Supplementary Table S2. To assess the robustness of the MR findings, diagnostic plots were generated. The scatter plots and funnel plots demonstrate consistent effect estimates with no substantial asymmetry, supporting the overall stability of the results. Additionally, leave-one-out sensitivity analysis revealed that the exclusion of any single SNP did not materially alter the causal estimates between the lipid traits and osteoporosis, indicating that the associations were not driven by outlier variants. Collectively, these findings affirm the reliability of MR analysis (Supplementary Figures S1 and S2).

### 3.2. Exploration of the Causal Effects of Immune Cell Characteristics on Osteoporosis

A total of 731 immune cell phenotypes were subjected to two-sample Mendelian randomization (MR) analysis to explore their potential causal associations with osteoporosis (Supplementary Table S3). To enhance the reliability of the findings, we applied the following stringent filtering criteria: (1) inverse-variance weighted (IVW) *p* < 0.01; (2) consistent effect direction across five MR models; and (3) no evidence of horizontal pleiotropy (MR-Egger intercept *p* > 0.05). After correcting for multiple comparisons, a subset of 15 immune cell traits exhibited significant associations with osteoporosis via the IVW method, as detailed in Table 1 and Supplementary Table S4. These included two B cell subsets, three conventional dendritic cell (cDC) populations, six T, B, and NK (TBNK) lymphocyte subtypes, three monocyte subpopulations, and one myeloid cell phenotype. Among these, one B immune cell was linked with increased osteoporosis risk: IgD− CD38dim %B cell (IVW: odds ratio [OR] 1.0524, 95% confidence interval [CI] 1.0253–1.0801; *p* = 0.000); the other B cells were linked with decreased osteoporosis risk: IgD+ CD38dim %lymphocyte (IVW: odds ratio [OR] 0.9702, 95% confidence interval [CI] 0.9503–0.9906; *p* = 0.004). Two cDC immune cells were linked with increased osteoporosis risk: HLA DR on plasmacytoid DC (IVW: odds ratio [OR] 1.0497, 95% confidence interval [CI] 1.0167–1.0837; *p* = 0.003), and HLA DR on DC (IVW: odds ratio [OR] 1.0718, 95% confidence interval [CI] 1.0253–1.1205; *p* = 0.002); the other cDC cell was linked with decreased osteoporosis risk: CD11c+ CD62L− monocyte %monocyte (IVW: odds ratio [OR] 0.9102, 95% confidence interval [CI] 0.8624–0.9735; *p* = 0.005). For TBNK the immune cells, four traits were linked with increased osteoporosis risk: HLA DR++ monocyte %leukocyte (IVW: odds ratio [OR] 1.1646, 95% confidence interval [CI] 1.0846–1.2506; *p* = 0.000), CD8br %leukocyte (IVW: odds ratio [OR] 1.1175, 95% confidence interval [CI] 1.0306–1.2118; *p* = 0.007), HLA DR+ CD4+ %lymphocyte (IVW: odds ratio [OR] 1.1500, 95% confidence interval [CI] 1.0596–1.2482; *p* = 0.001), and SSC-A on HLA DR+ T cell (IVW: odds ratio [OR] 1.0797, 95% confidence interval [CI] 1.0206–1.1421; *p* = 0.008), and two traits were linked with decreased osteoporosis risk: HLA DR+ NK AC (IVW: odds ratio [OR] 0.9251, 95% confidence interval [CI] 0.8741–0.9791; *p* = 0.007) and CD16− CD56 on NK (IVW: odds ratio [OR] 0.9515, 95% confidence interval [CI] 0.9178–0.9863; *p* = 0.007). For the monocyte immune cells, all traits were linked with increased osteoporosis risk: HLA DR on CD14+ CD16− monocyte (IVW: odds ratio [OR] 1.1029, 95% confidence interval [CI] 1.0651–1.1420; *p* = 0.000), HLA DR on CD14+ monocyte (IVW: odds ratio [OR] 1.1014, 95% confidence interval [CI] 1.0625–1.1418; *p* = 0.000), and HLA DR on monocyte (IVW: odds ratio [OR] 1.0562, 95% confidence interval [CI] 1.0181–1.0958; *p* = 0.004). For the myeloid immune cells, all traits were linked with increased osteoporosis risk: HLA DR on CD33br HLA DR+ CD14dim (IVW: odds ratio [OR] 1.0593, 95% confidence interval [CI] 1.0210–1.0991; *p* = 0.002).

### 3.3. Exploring the Relationship Between Lipidomes and Osteoporosis-Associated Immune Cells

**Figure 4:**
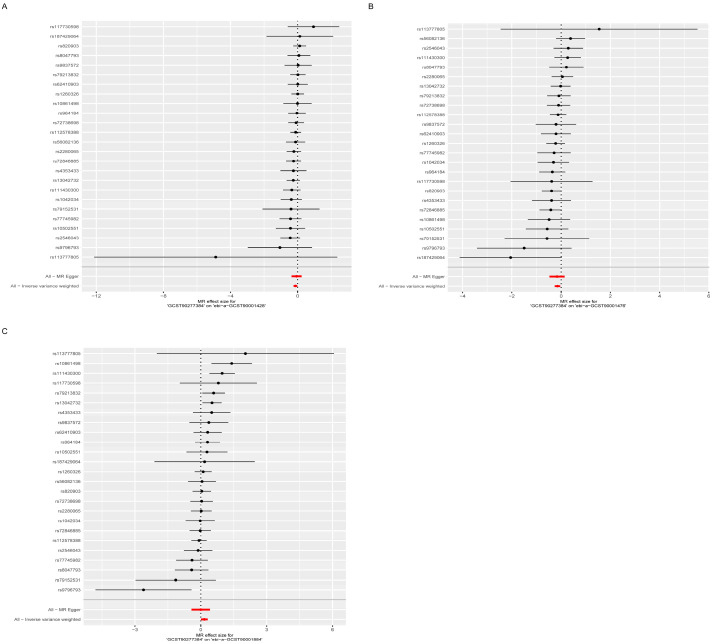
Forest plots illustrating the associations between triacylglycerol levels of 48:3 and immune cell phenotypes. (**A**) Higher plasma levels of triacylglycerol (48:3) were negatively associated with the proportions of IgD− CD38dim %B cells. (**B**) A negative association was observed between triacylglycerol levels of 48:3 and the proportion of HLA DR++ monocyte %leukocytes. (**C**) Triacylglycerol levels of 48:3 showed a positive association with CD16− CD56+ natural killer (NK) cell populations.

After identifying 15 immune cell subsets significantly associated with osteoporosis, we further evaluated their relationships with lipid metabolites through two-sample Mendelian randomization (MR) analysis. To ensure robustness, associations were retained based on the following criteria: (1) inverse-variance weighted (IVW) *p* < 0.05; (2) consistent effect direction across all five MR models; and (3) the absence of directional pleiotropy (*p* for MR-Egger intercept > 0.05). Based on these stringent thresholds, three immune cell–lipidome pairs demonstrated statistically robust and biologically meaningful associations. Triacylglycerol levels of 48:3 were negatively associated with IgD− CD38dim %B cell (IVW: β −0.1312, 95% confidence interval [CI] −0.2431–−0.0193; *p* = 0.020) (Figure 4A) and HLA DR++ monocyte %leukocyte (IVW: β −0.1512, 95% confidence interval [CI] −0.2653–−0.0371; *p* = 0.010) (Figure 4B), and positively associated with CD16− CD56+ on NK (IVW: β 0.1611, 95% confidence interval [CI] 0.0050–0.3171; *p* = 0.040) (Figure 4C). Heterogeneity and sensitivity analyses were performed on the results of the MR analysis of one lipidome associated with three immune cell features. The scatter plot and funnel plots also indicate the stability of the results, and the leave-one-out test showed that no SNP loci had a serious impact on the possible genetic relationship between the lipidome-associated loci and immune cells. Collectively, these findings validate the robustness of the MR analysis (Supplementary Figures S3–S5).

### 3.4. Immune Cell Characteristics Partly Mediate the Association Between the Lipidome and Osteoporosis

**Figure 5:**
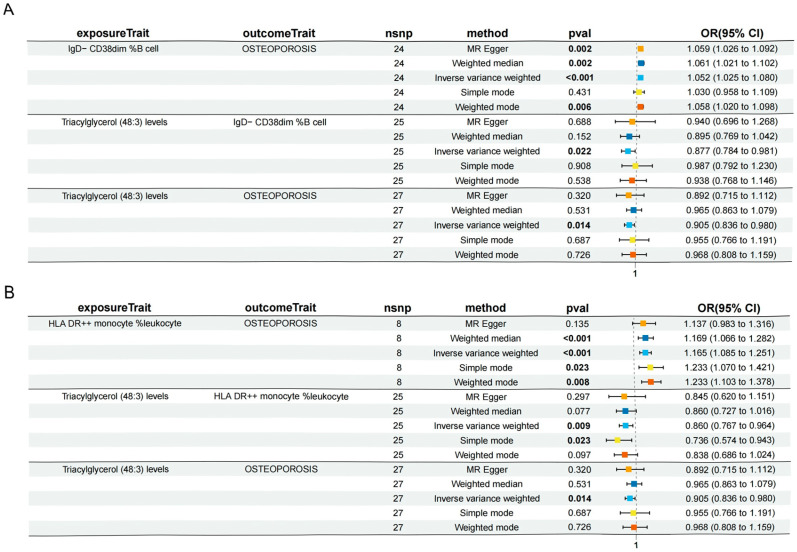
Forest plots illustrating the partial mediating effects of immune cell traits on the association between lipid metabolites (lipidome) and osteoporosis. (**A**) The proportion of IgD− CD38dim %B cells exhibited a negative mediating effect on the relationship between plasma triacylglycerol levels of 48:3 and osteoporosis risk. (**B**) A similar negative mediating effect was observed for HLA DR++ monocyte %leukocyte in the pathway linking triacylglycerol levels of 48:3 and osteoporosis and osteoporosis.

By assessing the mediation effect between the lipidome and osteoporosis through mediation analysis of immune cell characteristics, we found two group mediation effects with statistical significance, which illustrate the potential pathogenesis of osteoporosis (Figure 5). First, IgD− CD38dim %B cells partly negatively mediated the associations between triacylglycerol levels of 48:3 and osteoporosis. The mediating effect and proportion were −0.00669 and 6.73%, respectively (Figure 5A, Table 2), meaning that this immune cell attenuated the protective effect of triacylglycerol levels of 48:3 and accounted for 6.73% of the direct effect. Second, HLA DR++ monocyte %leukocyte partly negatively mediated the associations between triacylglycerol levels of 48:3 and osteoporosis; the mediating effect and proportion were −0.023 and 23.2%, respectively (Figure 5B, Table 2). Similarly, these immune cells exerted a facilitating effect by downregulating the triacylglycerol levels of 48:3 against osteoporosis, accounting for a greater proportion of the effect than the effect of IgD− CD38dim %B cells, with 23.2% of the direct effect.

## 4. Discussion

The pathological basis of osteoporosis is rooted in an imbalance of the bone remodeling process, marked by an increase in bone resorption by osteoclasts and a decrease in bone formation by osteoblasts, resulting in accelerated bone loss [17]. Osteoporosis is prevalent among older individuals, particularly postmenopausal women, and has emerged as a significant public health concern in the context of an aging global population. Current biochemical markers for the early clinical diagnosis of osteoporosis exhibit low sensitivity, highlighting the urgent need for novel biomarkers to predict the onset of bone loss. Recent research has implicated metabolites produced through cellular and tissue activities, as well as the immune microenvironment, in the pathogenesis of osteoporosis. Metabolites such as lipids, amino acids, carbohydrates, organic acids, and vitamins play key roles in various metabolic pathways, interacting with the neuroendocrine-immune system to create a complex, interrelated network. Lipids, which encompass a diverse array of compounds, such as glycerophospholipids, sphingolipids, and sterols, fulfill crucial biological roles, including the formation of cell membranes, energy storage, intracellular signaling, and the regulation of local hormones [18]. Recent studies have highlighted the significant biological functions of lipids and their derivatives as key mediators in bone physiology [19]. Reports have indicated that saturated fatty acids, such as palmitic acid, can diminish the functionality and viability of osteoblasts. In contrast, certain unsaturated fatty acids, including linoleic acid and palmitoleic acid, have been found to promote the differentiation, mineralization, and survival of osteoblasts [20,21], while also inhibiting the differentiation and activity of osteoclasts [22]. Hence, comprehending the impact of lipids and their distinct derivatives in modulating bone cell equilibrium is of paramount importance. Epidemiologic evidence suggests an association between osteoporosis and cardiovascular disease [23]. Previous studies have reported that cholesterol, triacylglycerol, and LDL are negatively correlated with spinal and systemic bone mass [24]. In a prospective study, serum cholesterol concentrations increased as spinal BMD decreased. Yi-Hsiang Hsu et al. showed that a significant negative correlation was found between whole-body BMD and standardized indices of cholesterol, triacylglycerol, and LDL concentrations in whole blood after adjusting for body weight, adiposity, and other confounders [25]. A similar pattern was found between whole hip BMC and serum lipids, but these were not significant. These results emphasize the importance of lipid profiles as protective/risk factors for osteoporosis and provide a theoretical basis for the further exploration of potential mechanisms.

The dynamic balance of the bone and bone marrow ecological niche is dynamically regulated by nutrients. Cardiovascular diseases are positively correlated with the risk of osteoporosis, suggesting that hyperlipidemia and/or hypercholesterolemia are closely related to bone metabolism. Cholesterol and its metabolites affect the dynamic balance of bone by regulating the differentiation and activation of osteoblasts and osteoclasts [26]. In a rat model, hyperlipidemia induced by a high cholesterol diet decreased alveolar bone density and increased the number of anti-tartrate acid phosphatase-positive osteoclasts. Low-density lipoprotein promoted osteoclastogenesis [27]. Compared to LDL, HDL is known as the “good” cholesterol and is an important component of the lipoprotein transport system, regulating plasma and tissue lipid metabolism and homeostasis [28]. Apolipoprotein A-I (ApoA-I) is the major protein component of plasma HDL particles. Consistent with the protective effect of HDL on bone metabolism, the addition of HDL can eliminate the effect of oxidized LDL on osteoblast apoptosis [29]. However, Chen et al. [30] proposed an opposing perspective, particularly in elderly populations and specific skeletal sites, such as the lumbar spine. Their findings suggest that high-density lipoprotein cholesterol (HDL-C) may exert negative effects on bone mass by either inhibiting osteoblast-mediated bone formation or promoting osteoclastic bone resorption, thereby contributing to decreased bone mineral density (BMD). Furthermore, they reported that triacylglycerols (TAGs), as a broad lipid category, may positively regulate bone density. However, their study did not delineate the impact of specific TAG subtypes on skeletal health. Building on this, Zhang et al. [31] provided causal evidence linking multiple circulating lipid fractions to site-specific bone loss using Mendelian randomization analysis. They demonstrated negative causal associations between very-low-density lipoprotein cholesterol (VLDL-C) and BMD at the calcaneus, lumbar spine, and total body. In addition, apolipoprotein B (ApoB) and the ApoB/ApoA1 ratio were significantly associated with reduced BMD at both the calcaneus and lumbar spine, while elevated HDL-C levels exerted the most extensive negative causal impact, being inversely associated with BMD at all measured skeletal sites. These results substantially reinforce the credibility of previously observed correlations by offering robust causal inference. Moreover, Zhou et al. [32], in a large-scale cohort of 3558 Chinese patients with osteoporotic fractures (OPF) undergoing surgical treatment, reported a significant positive association between serum TAG levels and lumbar spine BMD (β = 0.015, *p* = 0.033). Notably, this association was especially pronounced among older adults, and further stratified analyses revealed that TAG levels below 1.26 mmol/L were positively correlated with BMD, suggesting a dose-dependent protective effect within a physiological range.

These findings collectively suggest that TAGs may exert a bone-protective function, but only within a specific threshold concentration window. When TAG concentrations fall below this threshold, bone health may be compromised due to insufficient lipid availability for osteoblast function or energy metabolism. Conversely, excessively high TAG levels appear to confer no additional benefit and may even increase the burden of skeletal disorders, implying the presence of a “protective ceiling” effect in lipid–bone interactions. Among all plasma lipid classes, triacylglycerols are the most abundant, and their subclass-specific roles in bone metabolism remain largely unexplored. In our study profiling 179 plasma lipid species, we identified two TAG molecules with divergent associations with osteoporosis: Triacylglycerol levels of 48:0 were identified as a risk factor, whereas triacylglycerol levels of 48:3 showed a protective association with BMD. These novel findings not only support the emerging view of lipid–bone crosstalk but also provide mechanistic clues for the differential effects of lipid subtypes in skeletal health, offering potential targets for biomarker discovery and therapeutic intervention in osteoporosis.

The intricate relationship between immune cells and lipid metabolism has been increasingly elucidated by a growing body of authoritative studies. Immune cells can actively intervene in lipid metabolic processes through cytokine secretion and metabolic reprogramming. Notably, they possess the capacity to clear deleterious lipid metabolites, functioning as “metabolic scavengers” or “regulators”, particularly in the context of chronic inflammation, atherosclerosis, hepatic steatosis, and metabolic syndrome. You et al. [33] revealed that dendritic cells (DCs) serve as central metabolic signaling hubs, capable of sensing lipid overload or lipid peroxidation products and subsequently activating antioxidant and lipid-processing pathways via Nrf2 and PPARγ signaling. Additional investigations have highlighted the anti-inflammatory roles of M2 macrophages and regulatory T cells (Tregs), which secrete IL-10 and TGF-β to suppress lipid peroxidation-induced chronic inflammation and support tissue repair and metabolic homeostasis [34]. Strikingly, Lucas et al. [35] demonstrated that Tregs contribute to bone metabolic homeostasis by both suppressing osteoclastogenic factors (e.g., RANKL) and eliminating pro-inflammatory lipid species. In parallel, Zhang et al. [7] proposed that macrophages, via the expression of scavenger receptors CD36 and SR-A, internalize oxidized low-density lipoprotein (oxLDL) and, when polarized toward an M2 phenotype, secrete IL-10 and TGF-β, thereby dampening local inflammation and promoting osteoblast differentiation. These converging lines of evidence underscore the protective role of immune cells in lipid metabolism, indicating that they not only eliminate harmful lipid derivatives but also secrete regulatory mediators and undergo metabolic shifts that mitigate lipotoxicity-induced bone damage. Such mechanisms may play an essential role in preserving bone mass and counteracting osteoporosis.

Conversely, certain immune cells have been shown to compete with or neutralize bone-beneficial lipid species, ultimately undermining their osteoprotective effects. Under inflammatory conditions, M1-polarized macrophages become hyperactivated and may overutilize short-chain fatty acids (SCFAs) to drive NF-κB signaling, reducing the relative abundance of Tregs and promoting bone resorption [36]. Patel et al. [37] were among the first to report that saturated fatty acids activate TLR4 signaling in immune cells, inducing lipid peroxidase enzymes that degrade unsaturated lipids, thereby impairing osteoblastic function. These findings reflect a critical dimension of the immune–lipid interface, particularly relevant in chronic inflammation, autoimmune diseases, and age-related bone loss. They also align with our current results, which identify a positive association between circulating triacylglycerol levels of 48:3 and bone mineral density (BMD), suggesting a potential anabolic effect on bone. However, in individuals with elevated proportions of IgD− CD38dim %B cells and HLA DR++ monocytes, this osteoprotective association was markedly attenuated. We hypothesize that these two pro-inflammatory immune subsets may contribute to increased bone loss by secreting osteoclastogenic factors, promoting inflammatory cascades, or interfering with lipid metabolic pathways that would otherwise support skeletal integrity. These findings provide further evidence that a “lipid–immune–bone axis” antagonism may exist under certain pathological conditions.

“Osteoimmunology” is an emerging and rapidly evolving interdisciplinary field that encapsulates the complex crosstalk between the immune and skeletal systems. Recent research has significantly furthered our comprehension of this intricate interplay, with a notable focus on the influence of the immune system upon osteoclast activity. Osteocytes and immune cells originate from a shared lineage, specifically from multipotent progenitors (MPPs) [38]. These MPPs, which differentiate from hematopoietic stem cells (HSCs) located in the bone marrow, subsequently give rise to adaptive immune cells through common lymphoid progenitors (CLPs) and osteoclasts through common myeloid progenitors (CMPs) [39]. Activated T cells and B cells are able to secrete pro-osteoclastogenic factors [40,41], while interferon-γ promotes osteoblast differentiation, inhibits bone marrow adipocyte formation, and plays different regulatory roles at different stages of osteoclast differentiation [42]. The findings of bone immunology suggest a complex link between immune cells and bone metabolism, but many specific mechanisms have not yet been revealed. OP, as a systemic metabolic bone disease, occurs in close association with abnormalities of bone metabolism, but the potential for certain genetic associations between immune cells and OP to reflect the progression of OP and the strength of immune responses deserves further investigation. Our findings highlight a potential causal link between specific immune cell subsets, lipid metabolism, and bone mineral density, particularly through the modulation of triacylglycerol subclasses. This proposed “immune–lipid–bone axis” aligns with recent mechanistic insights from autoimmune bone diseases, such as rheumatoid arthritis (RA). Notably, Llorente et al. [43] emphasized that osteoporosis in RA is not merely a secondary comorbidity, but rather the result of complex immuno-skeletal interactions. In early RA, pro-inflammatory cytokines, such as IL-1, IL-6, IL-8, and TNF-α, actively induce osteoclastogenesis through both canonical RANK/RANKL/OPG signaling and alternative pathways, including the Wnt/DKK1/sclerostin-mediated suppression of osteoblast activity. Moreover, activated T cells and synovial fibroblasts upregulate RANKL expression, further enhancing osteoclast activation and promoting systemic bone loss. Importantly, this review also highlights the preclinical impact of autoantibodies, such as rheumatoid factor (RF) and anti-citrullinated protein antibodies (ACPAs), which contribute to bone erosion and systemic osteoporosis prior to the clinical onset of joint inflammation. These findings reinforce the hypothesis that immune dysregulation itself, even before overt disease, can serve as a causal driver of bone structural deterioration. In the context of our MR results, which link immune phenotypes and lipid subtypes to BMD changes, the evidence from RA pathogenesis provides a biological rationale for considering immune cell–lipid interactions as upstream regulators of bone remodeling, independent of traditional mechanical factors. The emerging concept that BMD may function as an early biomarker of immune-driven skeletal damage further underscores the need to investigate immune-metabolic markers in subclinical stages of bone disease.

The mediating effects of B cells in our study were characterized by two main molecular features involving immunoglobulin D (IgD) and CD38. However, CD19 on IgD− CD38dim %B cells was positively causally associated with OP, whereas IgD+ CD38dim %lymphocyte was negatively causally associated with OP. Immunoglobulin D (IgD) is a member of the immunoglobulin family, whose precise functional role remains a subject of ongoing debate. Recent studies [44] suggest that IgD may serve as an immunoregulatory protein, potentially involved in the maturation of B cells. However, its full range of functions and mechanisms of action are yet to be fully elucidated. Similarly, the function of CD38 is highly contentious. This molecule exhibits dual functionality, acting both as a receptor and as an enzyme. Its role in immune responses appears to be context-dependent, varying significantly with the specific disease, cell type, and animal model under investigation [45]. These complexities highlight the need for further research to clarify the precise contributions of both IgD and CD38 in immune regulation. Nigam et al. [46] demonstrated that the deficiency of CD38 accelerates immune processes, notably by enhancing the production of receptor activator of nuclear factor-κB ligand (RANKL) by CD27+ CD38− memory B cells. This, in turn, promotes the proliferation and differentiation of osteoclasts, ultimately contributing to alveolar bone damage. Similarly, Breuil et al. [12] observed a reduction in CD19+ CD27+ CD5− CD38+ memory B cells in postmenopausal women with OP. Currently, there is no direct evidence to establish a definitive causal relationship between IgD− CD38dim %B cells and IgD+ CD38dim %lymphocytes in the context of OP. We hypothesize that the complex and dynamic interactions between various B cell subsets may play a crucial role in the pathogenesis of OP, potentially modulating disease progression through distinct immune mechanisms. In our study, monocytes also play a role in the development of osteoporosis, meaning that we predicted a positive causal relationship between HLA DR++ monocyte %leukocyte and OP. Monocytes play a pivotal role in antigen presentation, a process that is primarily mediated by major histocompatibility complex class II (MHC II). MHC II serves as a crucial transcriptional coactivator and an essential regulator of osteoclast differentiation, further implicating monocytes in the pathogenesis of OP [47,48].

Studies have shown that RANKL (encoded by the Tnfsf11 gene) serves as an important cytokine in the formation of osteoclasts and B lymphocytes. It functions by signaling through RANK, which is expressed on osteoclast progenitors and B lymphocytes. Mice lacking RANK or RANKL demonstrated severe osteoporosis due to disrupted osteoclastogenesis, characterized by the diminished presence of mature B220+IgD+ B lymphocytes in both the spleen and lymph nodes [49]. Previous studies have shown a significant increase in the number and activity of lymphocyte lineage cells in individuals diagnosed with osteoporosis or bone loss, which is consistent with the results of our study that IgD− CD38dim %B cell is a risk factor in osteoporosis. IgD− CD38dim %B cell antagonizes (negatively mediates) the protective effect of triglyceride levels of 48:3 on osteoporosis. The interaction between immune cells and osteoblasts has redefined our understanding of the regulation of bone resorption. Furthermore, interactions between immune cell types are of particular importance under inflammatory conditions of dyslipidemia, and we reasonably propose, on the basis of our results, that HLADR++ monocytes are synergistically recruited to exert antagonistic effects. Macrophages, including osteoclasts, which arise from monocyte lineages, play a critical role in both the pathogenesis of osteoporosis and functions of the innate immune system. In the pro-inflammatory microenvironment associated with dyslipidemia, the monocyte–macrophage system is activated, with HLADR++ monocytes potentiating osteoclast formation. This effect may be facilitated by pro-inflammatory cytokines, notably IL-6 and TNF-α, which are established promoters of osteoclastogenesis [50]. Our research sheds light on the modifications in lipidome dynamics involved in bone remodeling, and further unravels the potential interactions between lipidome, immune cells, and bone tissue through Mendelian randomization analysis. These findings are potentially poised to contribute to the development of molecular therapies and nutritional approaches for osteoporosis management.

### Limitation

This study presents novel insights into the causal relationship between lipid metabolism and osteoporosis using a mediator-based Mendelian randomization (MR) framework, emphasizing the potential intermediary role of immune cells. Despite the strengths of this approach, several limitations warrant careful consideration. First, the robustness of MR findings is contingent on the strength and validity of the genetic instruments employed. The instrumental variables used in this study, although statistically significant, explain only a modest proportion of the variance in the exposure traits, which could affect the accuracy of causal effect estimates. Second, human lipid metabolism is regulated within a multifactorial and dynamic biological system, shaped by interactions among genetic architecture, environmental conditions, and individual lifestyle factors. Some of these variables remain unmeasured and could contribute residual confounding beyond the scope of MR correction. Moreover, the lipidomic data utilized may not encompass the full spectrum of biologically active lipid species, particularly those with low abundance or tissue-specific relevance, limiting the functional depth of metabolic inference in the context of osteoporosis. Addressing these limitations will require future research integrating advanced metagenomic and multi-omics strategies to systematically characterize lipid metabolites and elucidate their therapeutic potential in osteoporosis. Importantly, the osteoporosis cohort used in the Finnish dataset included both primary and secondary forms of the disease. Given the heterogeneous etiologies of secondary osteoporosis, ranging from chronic disease, drug use, endocrine dysfunction to systemic comorbidities, the inclusion of such cases introduces phenotypic heterogeneity. This may obscure causal interpretations by introducing variation in underlying biological mechanisms, particularly those unrelated to lipid metabolism. Subsequent investigations may benefit from subgroup analyses, the fine stratification of disease phenotypes, or the construction of more homogeneous cohorts to improve the internal consistency of the study and the robustness of causal inference. Lastly, as the sample population was predominantly of European ancestry, caution is warranted when extrapolating findings to diverse ethnic groups. Replication in multi-ethnic cohorts will be essential to assess the trans-ancestral consistency of the observed associations and ensure broader generalizability.

## 5. Conclusions

Drawing from our study, we have pinpointed and speculated two lipidomes as pivotal in the progression of osteoporosis. Triacylglycerol levels of 48:0 emerged as a potential risk factor, whereas triacylglycerol levels 48:3 appeared to serve a protective role. Furthermore, our findings suggest that the direct protective effect of triacylglycerol levels of 48:3 may involve a hindering effect of IgD− CD38dim %B cells and HLA DR++ monocytes, thus influencing the development of osteoporosis. Two immune cell populations attenuated the potential protective effect of triacylglycerol levels of 48:3 against osteoporosis by detailed mediated effect analysis, from IgD− CD38dim %B cells and HLA DR++ monocytes, which produced antagonistic effect shares of 6.73% and 23.2% of their respective direct effects. This insight underscores their potential as targets for developing interventions against osteoporosis within dyslipidemic conditions.

## Figures and Tables

**Table 1:** Causal effects of immune cells on osteoporosis.

Traits	Methods	Beta	Low	Up	*p*
IgD− CD38dim %B cell	Inverse-variance weighted	0.051	0.025	0.077	0.000
IgD+ CD38dim %lymphocyte		−0.030	−0.051	−0.009	0.004
CD11c+ CD62L− monocyte %monocyte		−0.087	−0.148	−0.027	0.005
HLA DR++ monocyte %leukocyte		0.152	0.081	0.224	0.000
CD8br %leukocyte		0.111	0.030	0.192	0.007
HLA DR+ CD4+ %lymphocyte		0.140	0.058	0.222	0.001
HLA DR+ NK AC		−0.078	−0.135	−0.021	0.007
CD16− CD56+ on NK		−0.050	−0.086	−0.014	0.007
HLA DR on CD14+ CD16− monocyte		0.098	0.063	0.133	0.000
HLA DR on CD14+ monocyte		0.097	0.061	0.133	0.000
HLA DR on monocyte		0.055	0.018	0.091	0.004
SSC-A on HLA DR+ T cell		0.077	0.020	0.133	0.008
HLA DR on plasmacytoid DC		0.048	0.017	0.080	0.003
HLA DR on DC		0.069	0.025	0.114	0.002
HLA DR on CD33br HLA DR+ CD14dim		0.058	0.021	0.094	0.002

**Table 2:** The mediating effects and proportions of immune cell characteristics that partially mediate the association between the lipidome and osteoporosis.

Lipidomes	Immune Cell	Outcome	Mediated Effect	Mediated Proportion
Triacylglycerol levels of 48:3	IgD− CD38dim %B cell	Osteoporosis	−0.00669 (−0.0214, 0.00805)	6.73% (21.6%, −8.09%)
Triacylglycerol levels of 48:3	HLA DR++ monocyte %leukocyte	Osteoporosis	−0.023 (−0.0434, −0.00266)	23.2% (43.7%, 2.67%)

## Data Availability

The GWAS public database numbers are noted in the text. The codes supporting the results of this study are available from the corresponding authors upon request.

## Supplementary Materials

The following supporting information can be downloaded at: https://www.mdpi.com/article/10.3390/diagnostics15101287/s1, Figure S1: Scatterplot, funnel plot and leave-one-out test of the correlation between triacylglycerol levels 48:3 and osteoporosis; Figure S2: Scatterplot, funnel plot and leave-one-out test of the correlation between triacylglycerol levels 48:0 and osteoporosis; Figure S3: Scatterplot, funnel plot and leave-one-out test of the correlation between triacylglycerol levels 48:3 and IgD− CD38dim %B cells; Figure S4: Scatterplot, funnel plot and leave-one-out test of the correlation between triacylglycerol levels 48:3 and HLA DR++ monocyte %leukocytes; Figure S5: Scatterplot, funnel plot and leave-one-out test of the correlation between triacylglycerol levels 48:3 and CD16− CD56+ natural killer (NK) cell populations; Table S1: Causal effects of lipidome on osteoporosis; Table S2: Causal effects of osteoporosis on lipidome; Table S3: SNP loci in immune cells; Table S4: Causal effects of immune cells on osteoporosis.
