# Supplementary figures and images for: Immune Cells as Mediators of Lipidome Influence on Osteoporosis: Evidence from a Mediation Analysis
Source: Diagnostics (Basel). 2025 May 20;15(10):1287. doi: 10.3390/diagnostics15101287 (PMC12109629; doi:10.3390/diagnostics15101287)

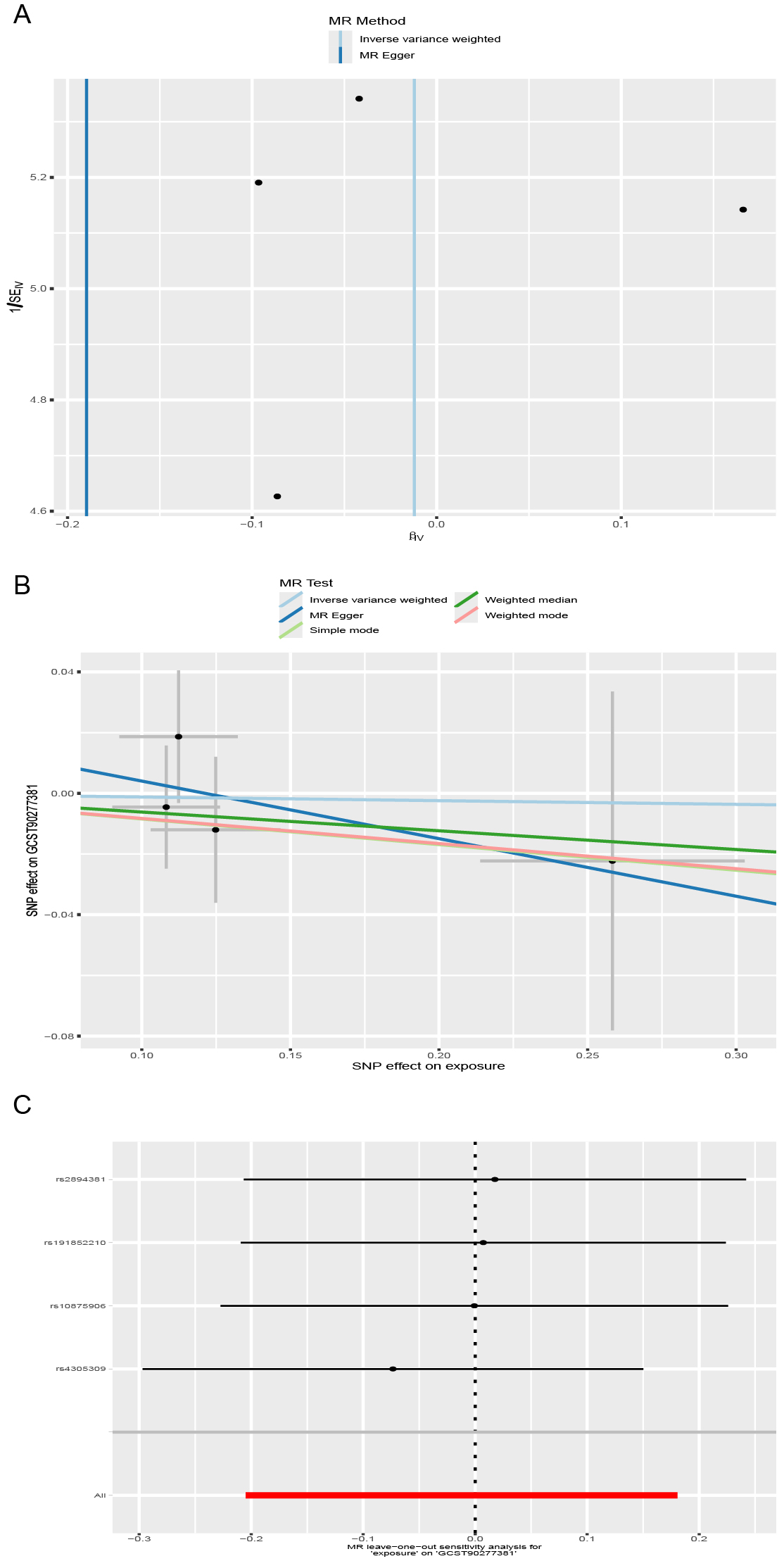

Supplement: Supplementary file 1 [file diagnostics-15-01287-s001.zip › Supplementary data files/Supplementary Fig. S1.tiff]

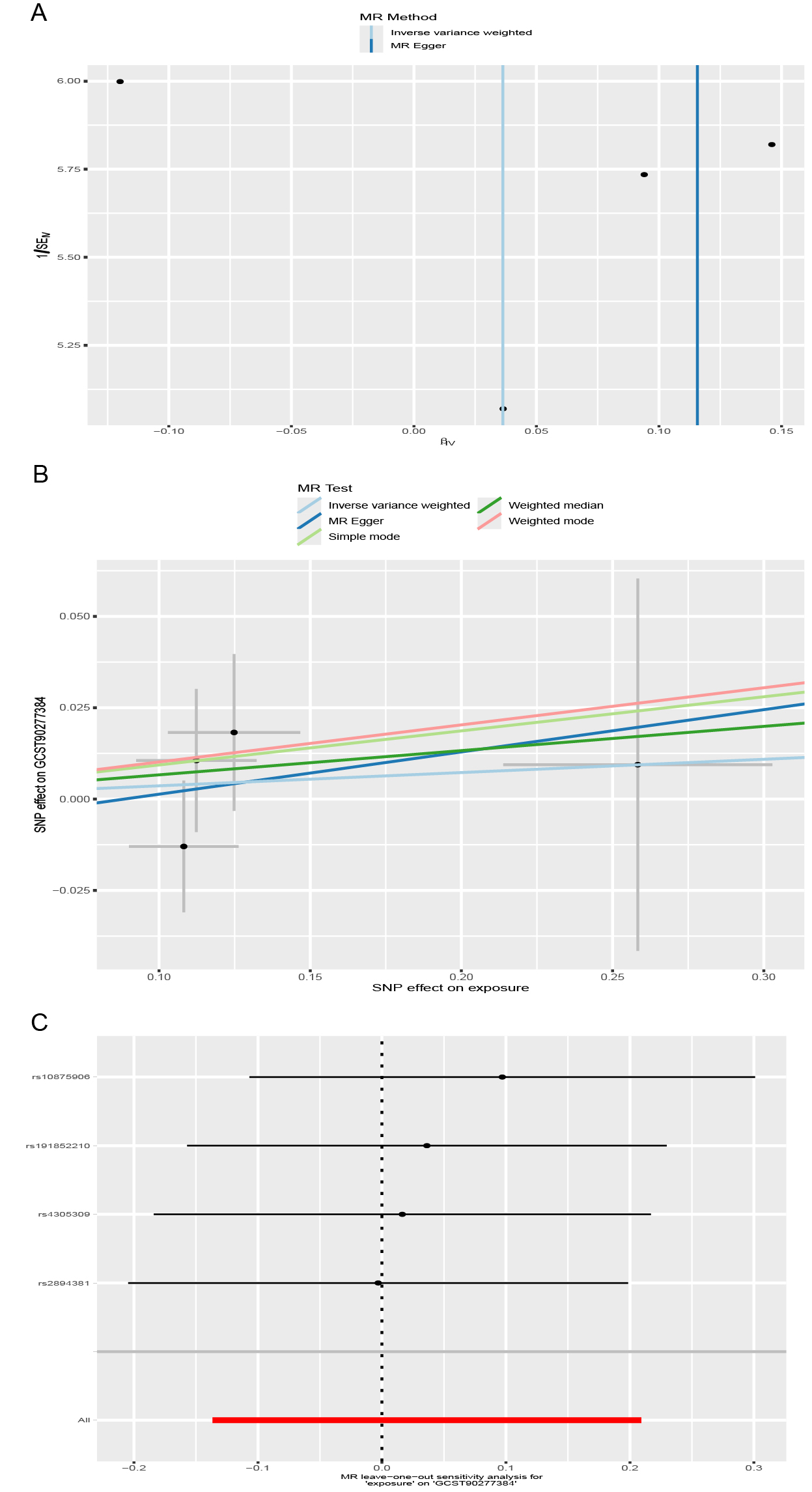

Supplement: Supplementary file 1 [file diagnostics-15-01287-s001.zip › Supplementary data files/Supplementary Fig. S2.tiff]

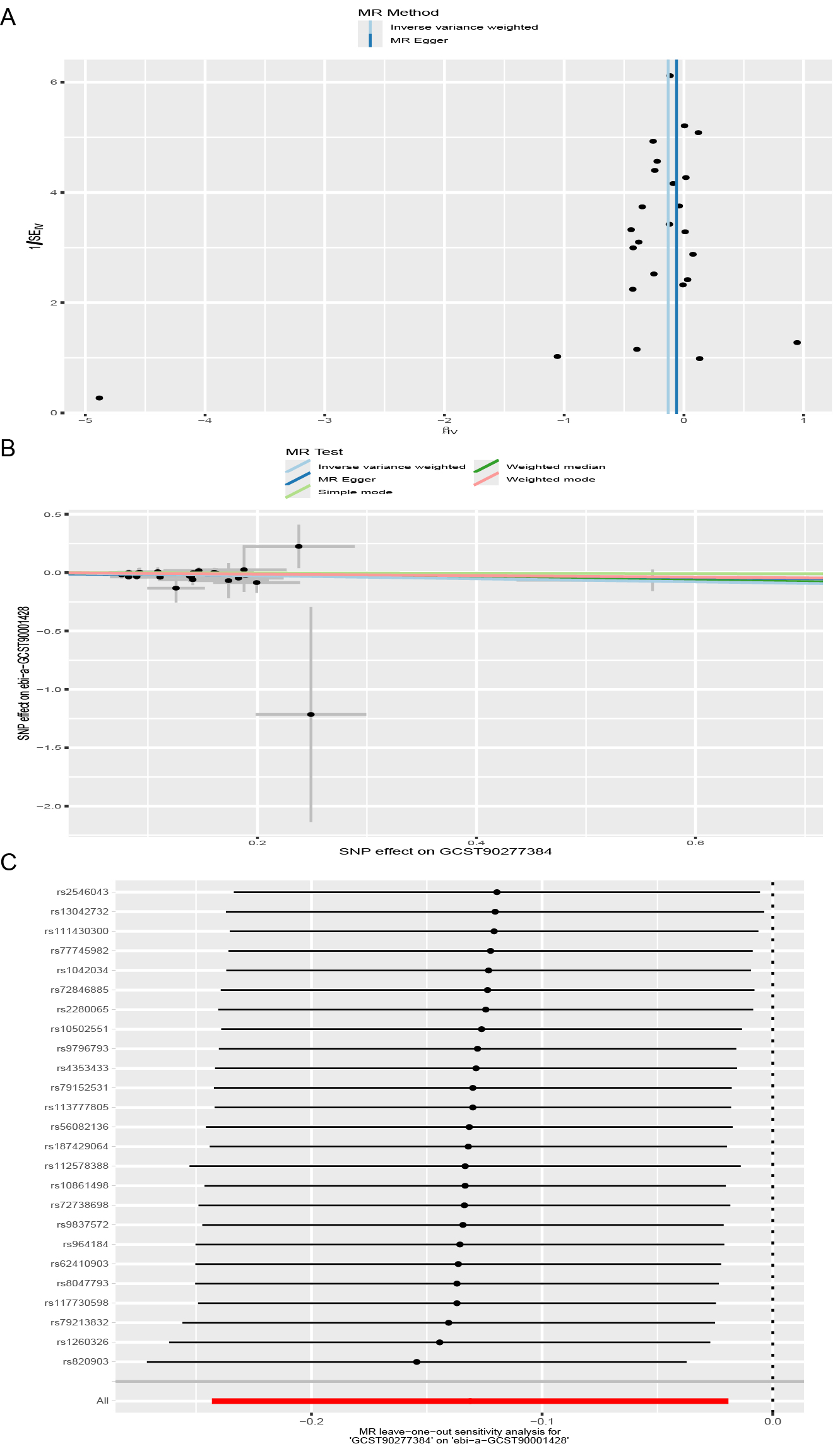

Supplement: Supplementary file 1 [file diagnostics-15-01287-s001.zip › Supplementary data files/Supplementary Fig. S3.tiff]

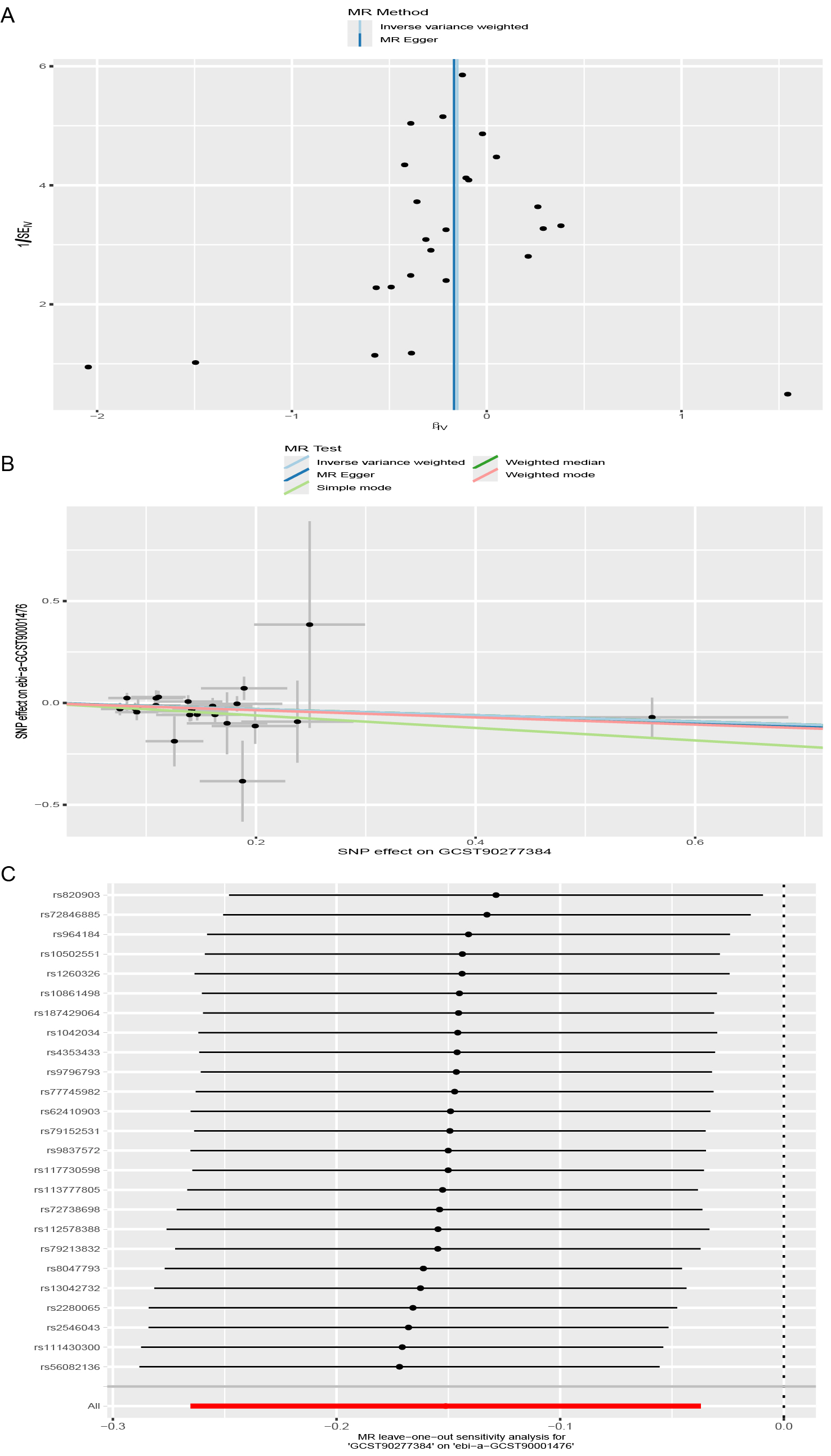

Supplement: Supplementary file 1 [file diagnostics-15-01287-s001.zip › Supplementary data files/Supplementary Fig. S4.tiff]

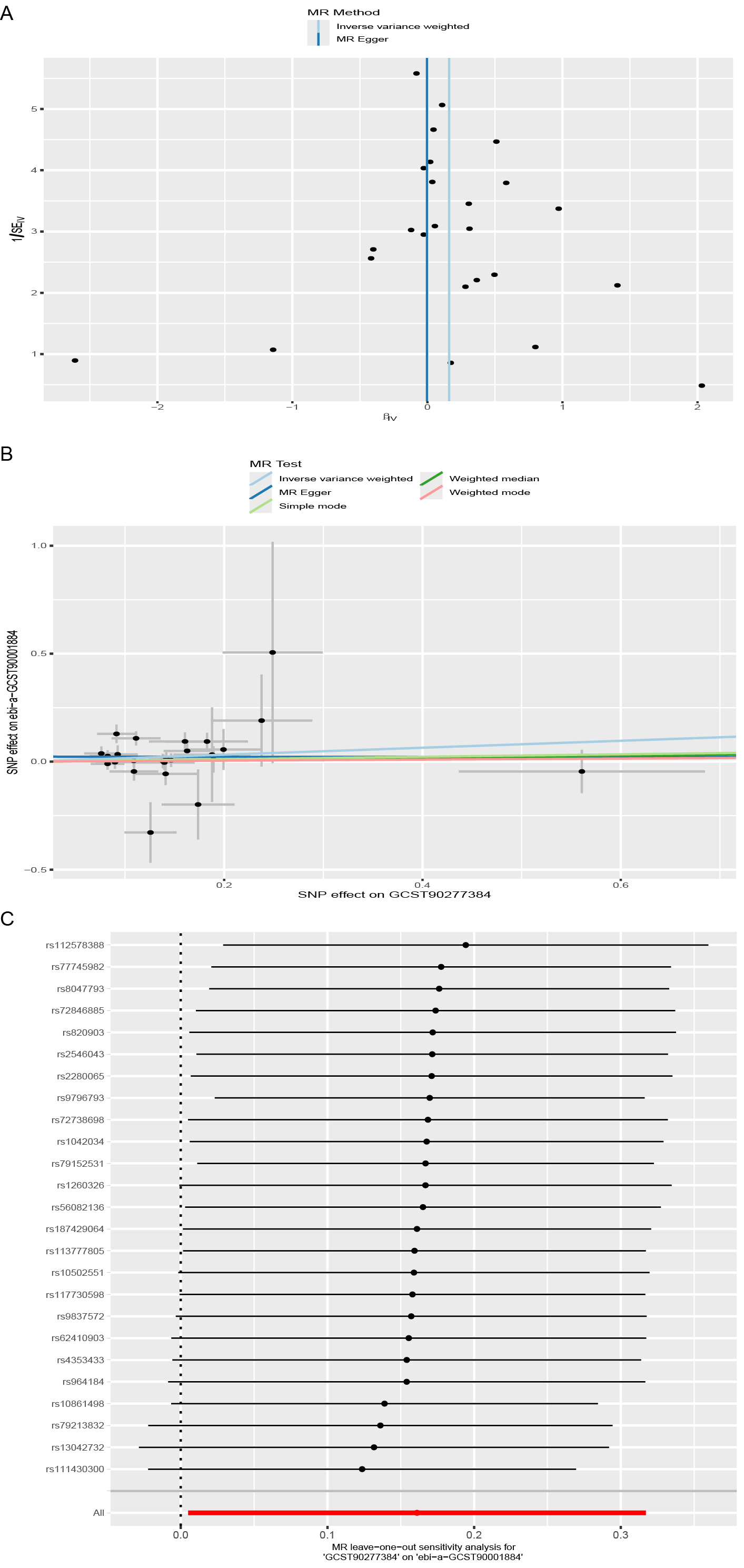

Supplement: Supplementary file 1 [file diagnostics-15-01287-s001.zip › Supplementary data files/Supplementary Fig. S5.tiff]
